# Natural Compounds in Prostate Cancer Prevention and Treatment: Mechanisms of Action and Molecular Targets

**DOI:** 10.3390/cells9020460

**Published:** 2020-02-18

**Authors:** Fabrizio Fontana, Michela Raimondi, Monica Marzagalli, Alessandro Di Domizio, Patrizia Limonta

**Affiliations:** 1 Department of Pharmacological and Biomolecular Sciences, University of Milano, 20133 Milano, Italy; fabrizio.fontana@unimi.it (F.F.); michela.raimondi@unimi.it (M.R.); monica.marzagalli@unimi.it (M.M.); alessandro.didomizio@unimi.it (A.D.D.); 2 SPILLOproject, 20037 Paderno Dugnano, Italy

**Keywords:** prostate cancer, natural compounds, phytochemicals, chemoprevention, novel therapeutic strategies

## Abstract

Prostate cancer (PCa) represents a major cause of cancer mortality among men in developed countries. Patients with recurrent disease initially respond to androgen-deprivation therapy, but the tumor eventually progresses into castration-resistant PCa; in this condition, tumor cells acquire the ability to escape cell death and develop resistance to current therapies. Thus, new therapeutic approaches for PCa management are urgently needed. In this setting, natural products have been extensively studied for their anti-PCa activities, such as tumor growth suppression, cell death induction, and inhibition of metastasis and angiogenesis. Additionally, numerous studies have shown that phytochemicals can specifically target the androgen receptor (AR) signaling, as well as the PCa stem cells (PCSCs). Interestingly, many clinical trials have been conducted to test the efficacy of nutraceuticals in human subjects, and they have partially confirmed the promising results obtained in vitro and in preclinical models. This article summarizes the anti-cancer mechanisms and therapeutic potentials of different natural compounds in the context of PCa prevention and treatment.

## 1. Introduction

Globally, prostate cancer (PCa) is the most frequently diagnosed tumor in men, being particularly common in Western countries [1]. In about 90% of cases, PCa is still organ-confined or only locally advanced at diagnosis, which makes it effectively treatable with prostatectomy or local radiotherapy. However, 30–40% of patients usually experience progression of disease [2]; at this stage, where tumor growth depends on androgens, the most effective treatment is represented by androgen-deprivation therapy, aimed at blocking hormone secretion and/or activity. This therapy is based on pharmacological castration, obtained by administration of GnRH agonists, alone or in combination with antiandrogens [3,4]; more recently, two major clinical trials, CHAARTED and STAMPEDE, have also demonstrated benefits of early initiation of chemotherapy concomitantly with hormonal therapy [5,6]. However, despite a good initial response, relapse occurs in the majority of patients within 2–3 years, and the tumor progresses towards a condition of resistance to castration [7]. Improved therapeutic options for castration-resistant patients are needed, since taxane-based (i.e., docetaxel) treatment and immunotherapy, as well as the novel therapies with enzalutamide and abiraterone, generally offer a progression-free survival of a few months [8,9]. Parallelly, bone metastases, occurring in 80% of advanced PCas and usually treated with radiation therapy and chemotherapy, are associated with considerable morbidity, adversely affect quality of life and several skeletal-related events [4,10]. Therefore, in the last years natural compounds have gained a lot of interest, due to their various anti-cancer effects. In fact, accumulating evidence has highlighted that nutraceuticals can exert growth-suppressing, pro-death, anti-metastatic, and anti-angiogenic activity in PCa cell lines and xenografts, while sparing normal prostate epithelial cells [11]. In particular, several mechanisms are involved in the anti-PCa actions of these molecules, including inhibition of androgen receptor (AR) axis and targeting of cancer stemness [12,13]. This review is aimed at summarizing the recent evidence about the role of different nutraceuticals in PCa prevention and therapy.

## 2. Natural Compounds with Potential to Treat Prostate Cancer

**Figure 1:**
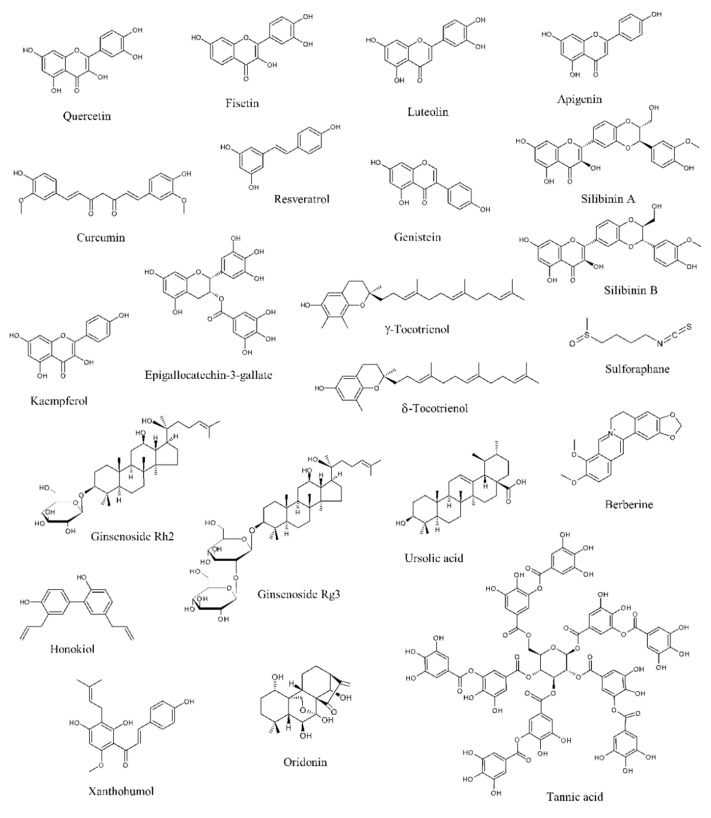
Chemical structures of the major anti-prostate cancer (PCa) phytochemicals.

Data from literature have pointed out that several natural products can selectively target numerous molecules and signaling pathways implicated in tumor development and progression [11,12,13]. Many of them have been tested in in vitro and in vivo studies, while some clinical trials have been conducted or are currently ongoing [11,12,13]. Among these naturally occurring molecules, quercetin, fisetin, luteolin, apigenin, curcumin, resveratrol, genistein, silibinin, kaempferol, epigallocatechin-3-gallate (EGCG), tocotrienols, sulforaphane, ginsenosides, ursolic acid, berberine, honokiol, xanthoumol, oridonin, and tannic acid have shown outstanding potential as anti-PCa agents in in vitro and preclinical experiments (Figure 1).

### 2.1. Natural Compounds Modulating the Androgen Receptor Axis

A number of studies indicates that PCa growth and progression are driven by the AR, a ligand-dependent transcription factor and member of the nuclear receptor family [14]. The AR is encoded by the *AR* gene located on the X chromosome at Xq11-12 and displays a N-terminal regulatory domain, a DNA-binding domain (DBD), a ligand-binding domain (LBD), and a C-terminal domain. In the absence of androgens, particularly dihydrotestosterone (DHT) and testosterone, it is complexed with chaperone proteins, heat-shock protein 90 (Hsp90) and 70 (Hsp70), in the cell cytoplasm. Upon ligand binding, it is transferred to the nucleus, where it homodimerizes due to the interactions of dedicated motifs in the DBD and in the LBD. Then, the dimerized receptor recognizes cognate DNA response elements in regulatory regions located in proximal or more distal intra- and inter-genic regions of androgen target genes [15,16]. It then recruits different coregulator proteins and epigenetic factors to generate a transcriptionally active complex able to upregulate downstream pro-survival gene expression [14].

Given its fundamental role in PCa cell proliferation, the AR signaling represents a crucial target for PCa management. In this context, pharmacological castration obtained via androgen-deprivation therapy is currently the most effective strategy for PCa treatment. However, PCa often becomes castration resistant [8,9]. One of the mechanisms underlying this change is an enhanced AR expression in the tumor cell. In particular, it has been shown that 28% of cancers resistant to androgen-deprivation therapy display AR upregulation due to amplification of its gene [17]. Another mechanism responsible for PCa androgen-independent growth is ligand promiscuity, caused by mutations of the *AR* gene that lead to amino acid substitutions in the LBD and subsequent decrease in the specificity and selectivity for ligands: the most common of them are T877A, F876L, W741L, and L701H. These mutant AR proteins bind to other steroids, including progesterone, estrogens, and glucocorticoids, which can activate the AR signaling pathway and promote PCa progression [18]. AR activation via ligand-independent mechanisms represents the third mechanism of androgen-independent PCa development [19]. Indeed, it has been found that tyrosine kinase receptor-activating ligands, such as epidermal growth factor (EGF) and insulin-like growth-factor-1 (IGF-1), can activate the AR through the phosphoinositide 3-kinase (PI3K)/Akt/mammalian target of rapamycin (mTOR) pathway [20,21,22,23,24]. Finally, various AR splice variants lacking the LBD have been recently reported: the AR N-terminal domain becomes constitutively active in the absence of the LBD, thereby promoting castration resistant proliferation [25,26].

Interestingly, various phytochemicals have been shown to modulate AR expression and activity.

Quercetin is a penta-hydroxylated flavonol, naturally occurring in tea, onions, apples, tomatoes, and capers and endowed with important chemopreventive and anti-cancer properties [27]. Yuan et al. demonstrated that in LNCaP PCa cells a protein complex containing the AR, specific protein 1 (Sp1) and c-Jun was generated in response to quercetin treatment and suppressed AR function. This resulted in the inhibition of the production of the prostate-specific, androgen-related tumor markers prostate-specific antigen (PSA) and human kallikrein-2 (hK2), as well as in the downregulation of androgen-related genes, such as ornithine decarboxylase (ODC) and NKX3.1 [28,29,30,31]. Interestingly, quercetin was also able to repress the expression of the AR splice variant 7 (AR-V7), which correlates to resistance to enzalutamide and poor prognosis, via Hsp70 inhibition [32].

Fisetin, a flavonol present in strawberries, apples, persimmons, onions, kiwi, and cucumbers, has been recently demonstrated to exert not only potent neuroprotective effects but also different anti-tumor activities [33,34]. In PCa, it was shown to specifically bind to the AR LBD. This interaction resulted in a decreased AR stability and amino-terminal/carboxyl-terminal (N-C) interaction, leading to a reduced transactivation of AR target genes. Moreover, fisetin treatment of LNCaP cells was followed by a downregulation of AR levels, due to a reduction in its promoter activity and to an increase of its degradation. In this cell line, the flavonol also synergized with bicalutamide in promoting apoptotic cell death. Finally, in AR-positive CWR22υ1 PCa cell-bearing mice, fisetin inhibited tumor growth and decreased PSA serum levels, suggesting that this compound is able to suppress AR activity also in vivo [35].

Luteolin, a flavone abundant in rosemary, thyme, parsley, broccoli, and celery, is characterized by anti-inflammatory, neuroprotective, and anti-cancer activity [36,37]. It was observed to induce a dose- and time-dependent decrease in AR mRNA and protein expression, as well as of intracellular and secreted PSA levels, in PCa cells. In particular, it appears to promote the AR-Hsp90 complex dissociation, causing AR degradation via the proteasome-ubiquitin pathway [38].

Curcumin is a polyphenol extracted from turmeric (*Curcuma longa*), which has shown great therapeutic potential [39,40,41]. This compound was demonstrated not only to decrease the expression of AR and AR-related cofactors, such as activator protein-1 (AP-1), nuclear factor kappa-light-chain-enhancer of activated B cells (NF-κB), CREB-binding protein (CBP), and NKX3.1, but also to reduce testosterone production in PCa cell lines and xenografts. This reduction in testosterone levels was associated with a downregulation of steroidogenic acute regulatory proteins, including cytochrome P450 11A1 (CYP11A1) and 3-beta-hydroxysteroid dehydrogenase 2 (HSD3B2), and in an enhanced expression of aldo-keto reductase 1C2 (AKR1C2), a 3-ketosteroid reductase responsible for the elimination of 5alpha-DHT and subsequent inactivation of AR [42,43,44,45].

Resveratrol is a grape-derived polyphenol that possesses numerous health benefits, including various chemopreventive effects [46]. It was found to target the AR axis in different in vitro and in vivo PCa models [47,48,49,50,51]. On one hand, in LNCaP cells it inhibited β-catenin nuclear translocation through hypoxia-inducible factor 1-α (HIF-1α) downregulation, thus suppressing β-catenin-mediated AR signaling [52]; similarly, it also repressed interleukin-6 (IL-6)-induced AR transcriptional activity [53]. On the other hand, in 22RV1 cells it promoted the AR splice variant ARV7 proteasomal degradation, by enhancing its polyubiquitination. These data indicate that resveratrol could be used not only for the treatment of androgen-responsive PCa but also for the management of the ARV7-positive castration-resistant tumor [54].

Genistein is a common phytoestrogen that can be obtained from soybeans [55]. Indeed, it was shown to inhibit the AR signaling via estrogen receptor-β (ER-β) and estrogen-related pathways, as well as through suppression of Akt/Forkhead box O3a (FOXO3a)/glycogen synthase kinase 3β (GSK-3β) and histone deacetylase 6 (HDAC6)-Hsp90 function, needed to stabilize the AR [56,57,58,59]. Notably, in a recent study by Mahmoud et al., genistein was also demonstrated to bind to both the wild and the T877A-mutant types of AR, specifically competing with androgens. In particular, while it suppressed proliferation of AR wild-type LAPC-4 cells, it exerted a dual role in T877A-mutated LNCaP and PC3 cell lines, by stimulating cell growth at lower doses and inducing cell death at higher concentrations [60]. Finally, in PCa cells genistein downregulated prostate androgen-regulated transcript-1 (PART-1) gene expression induced by DHT, thus affecting cell proliferation [61].

Other natural products that have been demonstrated to trigger similar inhibitory effects on the AR axis are sulforaphane [62,63,64,65], epigallocatechin-3-gallate (EGCG) [66,67], ginsenosides [68,69,70,71], silymarin [72], berberine [73], honokiol [74], and celastrol [75].

### 2.2. Natural Compounds Affecting Proliferation

Numerous natural compounds have been reported to exert growth-suppressive and anti-proliferative activities in PCa cells and xenografts.

Epidermal growth factor receptor (EGFR) is a receptor tyrosine kinase whose activation is associated with an increase in cell growth and survival, which explains why EGFR is commonly overexpressed/overactivated in tumors of epithelial origin, including PCa. In particular, after binding to its specific ligands, such as EGF and transforming growth factor α (TGFα), it triggers several downstream signaling pathways, including PI3K/Akt/mTOR, mitogen-activated protein kinases (MAPKs), Hedgehog (Hh) signaling, and NF-κB [76]. Many phytochemicals, including quercetin, luteolin, resveratrol, genistein, and berberine, have been shown to reduce EGFR levels, as well as to suppress its intrinsic tyrosine kinase activity and its ligand-induced activation, in different PCa cell lines and in vivo models [77,78,79,80,81].

The IGF axis is a complex signaling network implicated in different tumorigenic processes, particularly in cancer proliferation, survival, and metabolism. It involves the interaction between the peptide-ligands IGF1 and IGF2 and the receptors IGF1R and IGF2R, and its activation elicits downstream signals, such as the PI3K/AKT and the MAPK pathways [82]. Interestingly, the IGF axis represents a major target for the anti-PCa action of silibinin, a flavonoid endowed with antioxidant properties commonly found in the milk thistle (*Silybum marianum*) [83,84]. Indeed, it decreased IGF1 expression and increased IGFBP-3 levels in transgenic adenocarcinoma of the mouse prostate (TRAMP) models, thus inhibiting tumor growth and progression [85,86,87]. Similar results were also obtained after treatment of PCa-bearing mice with luteolin [88].

Emerging evidence has highlighted the key role played by the PI3K/AKT pathway in the development of castration resistant PCa. This cascade, which is activated in most of advanced PCas, acts as a fundamental driver for tumor cell proliferation, thereby allowing cancer cells to survive to the androgen deprivation-related cytotoxicity. Moreover, preclinical studies have highlighted a strict correlation between the PI3K/AKT and AR axes, evidencing a dynamic cross-talk between these cascades in the acquisition of androgen-deprivation therapy resistance. Therefore, there is an evident rationale for the development of novel PI3K inhibitors, which may be able to block castration-resistant PCa growth and survival [89]. In this setting, the interest in natural products has recently increased, due to their ability to specifically target the PI3K/AKT cascade. In particular, quercetin, apigenin, curcumin, genistein, sulforaphane, and EGCG have been demonstrated to attenuate PCa cell growth by downregulating this signaling pathway [90,91,92,93,94,95,96,97,98].

During PCa progression, both tumor invasion and chemoresistance are promoted by NF-κB. Indeed, constitutive activation of this protein has been commonly found in primary PCas and it is associated with AR loss and castration-resistant features. Thus, NF-κB is an important target for PCa management, owing to its role in tumorigenesis and therapy resistance [99]. Notably, downregulation of this protein and of its target genes has been highlighted after resveratrol, genistein, sulforaphane, ursolic acid, tocotrienol, and celastrol treatment [100,101,102,103,104,105].

Hh pathway activation is implicated in the development of different types of tumors, including PCa. In particular, many studies have pointed out that this signaling plays a crucial role in the progression of PCa to more aggressive and chemoresistant states [106]. Slusarz et al. demonstrated that seven common nutraceuticals, (i.e., genistein, curcumin, EGCG, resveratrol, apigenin, baicalein, and quercetin) can suppress the Hh pathway both in vitro and in vivo, with four of them (i.e., genistein, curcumin, resveratrol, and EGCG) decreasing not only Hh effector Gli1 expression but also Gli1 reporter activity [107].

Genome sequencing and gene expression analyses have evidenced the importance of the Wnt pathway in the development of castration resistant PCa [108]. Wnt signaling is also implicated in the cross-talk with the PCa microenvironment, where this protein is secreted by the tumor stroma and promotes therapy resistance, as well as in PCa stem cell self-renewal or expansion [109]. Preclinical studies have illustrated the potential of Wnt inhibitors in preventing PCa progression. Some of them have already been tested in phase I trials, although they have not been administered to PCa patients yet [108,109]. Interestingly, treatment of PCa cells with quercetin, curcumin, genistein, and silibinin resulted in growth suppression through Wnt cascade modulation [110,111,112,113].

MicroRNAs (miRNAs) are endogenous, ≈22 nucleotides, non-coding RNAs able to induce both transcriptional and translational arrest, thus functioning as either oncogenes or oncosuppressors, depending on the specific tumor type [114]. Concerning PCa, genistein has shown promise in modulating the levels of different oncogenic (i.e., miR221, miR222, miR151, and miR1260b) and oncosuppressor (i.e., miR-574-3p and miR34a) miRNAs, thus affecting cancer cell proliferation [115,116,117,118,119,120]. Similar encouraging data were also obtained from in vitro studies with luteolin, curcumin, resveratrol, ginsenoside Rh2, and celastrol [121,122,123,124,125,126].

### 2.3. Natural Compounds Inducing Canonical and Non-Canonical Cell Deaths

Apoptosis is commonly induced in PCa cells and xenografts treated with phytochemicals. In particular, many natural products have been found to trigger both the extrinsic and intrinsic apoptotic pathways, by activating cell surface death receptors, altering Bax/Bcl-2 ratio, increasing p21 levels and triggering caspase-8, -9, -3, and poly (ADP-ribose) polymerase (PARP) cleavage [127,128,129,130,131,132,133,134,135,136,137,138,139,140,141,142,143,144,145,146,147,148,149,150,151,152,153]. In this setting, proteostasis disruption appears to play a key role in the modulation of the nutraceutical-related apoptotic cell death. Indeed, while apigenin, luteolin, genistein, and celastrol inhibited the proteasomal activity and caused ubiquitinated protein accumulation in different PCa cell lines [154,155,156], quercetin, curcumin, silibinin, and tannic acid induced endoplasmic reticulum (ER) stress [157,158,159,160], a condition where unfolded/misfolded proteins accumulate in the ER lumen and promote the activation of distinct pro-death cascades, including the double-stranded RNA-dependent protein kinase PKR-like ER kinase (PERK)/eukaryotic initiation factor 2α (eIF2α)/activating transcription factor 4 (ATF4)/C/EBP homologous protein (CHOP) pathway and the inositol-requiring enzyme 1α (IRE1)/c-Jun N-terminal kinase (JNK)/p38 MAPK cascade [161]. Notably, curcumin- and silibinin-mediated ER stress was associated with generation of reactive oxygen species (ROS) and redox homeostasis alteration [159,162], which were also observed in resveratrol- and sulforaphane-treated PCa cells [163,164,165,166].

Interestingly, apoptotic cell death is not the only death mode triggered by natural compounds.

Berberine is a benzylisoquinoline alkaloid commonly found in the plants of the genus *Berberis* [167,168]. In a recent study by Zhang et al., it was shown to induce programmed necrosis in LNCaP and PC-82 PCa cell lines. In particular, mitochondrial protein cyclophilin-D (Cyp-D) was observed to be crucially involved in the modulation of berberine-related necrotic cell death. Indeed, berberine treatment resulted in ROS production, which promoted p53 translocation to mitochondria and its interaction with Cyp-D to open the mitochondrial permeability transition pore (mPTP), ultimately leading to necrosis induction [169]. Pro-necrotic effects were also exerted by curcumin in DU145 cells [170].

Paraptosis is a programmed cell death mode characterized by cytoplasmic vacuolation, particularly by ER dilatation and mitochondrial swelling [171,172]. Recently, we have demonstrated that δ-tocotrienol, a vitamin E derivative particularly abundant in annatto seeds, rice bran, and palm oil [173,174], can trigger both apoptosis and paraptosis in PC3 and DU145 cell lines. The mechanisms underlying its pro-paraptotic effects were found to correlate with activation of JNK and p38, as well as with proteotoxicity, since not only the protein synthesis inhibitor cycloheximide but also the ER stress inhibitor salubrinal successfully prevented the cytoplasmic vacuolation evoked by the treatment with this natural compound [175]. Similarly, paraptosis-like cytoplasmic vacuolation was also observed in celastrol-treated PC3 cells [176].

Autophagy is an evolutionarily conserved catabolic process generally used by the cell to eliminate cytoplasmic material, including misfolded proteins and damaged organelles, via lysosomal degradation: it involves the formation of double-membrane vesicles, the autophagosomes, that promote cytoplasmic cargo recycling after fusion with lysosomes, and it is regulated by different proteins, particularly by microtubule-associated proteins 1A/1B light chain 3B, commonly called LC3 [177]. It is now well known that autophagy can act as both tumor promoter and suppressor. The dual role of this mechanism in cancer cells apparently depends on tumor type, stage, and genetic context. Indeed, while on one hand the autophagic flux clearly suppresses tumorigenesis, on the other hand it acts as a key survival mechanism in response to stress, thus promoting cancer cell proliferation. In the context of PCa, curcumin, sulforaphane, silibinin, ursolic acid, honokiol, and oridonin triggered cytoprotective autophagy [178,179,180,181,182,183,184]; on the contrary, fisetin, resveratrol, and celastrol treatment resulted in autophagic cell death [185,186,187]. In particular, the fisetin- and resveratrol-mediated autophagic flux was associated with Akt/mTOR signaling pathway downregulation and AMP-activated protein kinase (AMPK) activation [185,186]. The autophagy induced by celastrol, a pentacyclic triterpenoid extracted from *Tripterygium Wilfordi* roots [188], was instead correlated to suppression of AR/miR-101 cascade [187].

### 2.4. Natural Compounds Impairing Metabolism

Tumor metabolism is usually characterized by a high flux of glucose through glycolysis and the pentose phosphate pathway, thus representing an important pharmacological target. Indeed, treatments aimed at blocking these pathways and/or shifting lactic acid fermentation towards mitochondrial oxidative phosphorylation have shown promise in reducing tumor growth [189]. In recent studies by Fonseca J et al., resveratrol was found to promote a shift towards mitochondrial oxidation in PCa cells concomitantly with the suppression of proliferation, and when this change was prevented by culturing tumor cells in glucose-free medium or via prolyl hydroxylase (PHD) inhibition-mediated stabilization of HIF-1α, the phenol did not affect oxidative phosphorylation and cell growth, indicating that the metabolic shift from glucose fermentation to oxidation is fundamental for its anti-cancer effects [190,191].

As mentioned above, cancer cells need an increase in glucose uptake to satisfy their high demand for cell growth and proliferation. This is mediated by glucose transporters (GLUTs) by a mechanism of facilitated diffusion. Fourteen different GLUT receptors (GLUT1-12, GLUT14, and H/*myo*-inositol transporter) exist: the enhanced glucose consumption observed in tumor cells has been associated with overexpression of GLUT1, commonly found in brain and erythrocytes, but may also involve other GLUTs, including the heart-, skeletal muscle-, and adipose tissue-specific GLUT4 [192]. Gonzalez-Menendez et al. showed that GLUT1 and 4 proteins are expressed in LNCaP and PC3 cells and that apigenin and phloretin are able not only to reduce glucose uptake but also to modify GLUT levels in these cell lines [193].

Phosphoglucomutase 3 (PGM3) belongs to the hexose-phosphate mutase family, and it mediates the conversion of glucose-1-phosphate to glucose-6-phosphate, thus regulating glycolysis and pentose phosphate shunt [194]. Recently, it has been demonstrated to be a specific target for the anti-PCa activity of sulforaphane [195], an organic isothiocyanate derived from broccoli and other cruciferous plants [196,197].

In the last decade, the metabolic rewiring underlying tumor increased proliferation has been reported to not only involve glucose metabolism but also lipid synthesis. The crucial role played by lipids in tumor progression has been evidenced by different studies demonstrating that normal cells, except for adipocytes and hepatocytes, uptake the fatty acids necessary for their growth from the diet; however, in tumor cells lipids are mostly obtained via de novo lipogenesis. In the case of PCa, many studies have highlighted that its precursor lesions are characterized by elevated endogenous lipogenesis, regardless of the levels of extracellular/circulating lipids [198,199,200]. The increased de novo lipogenesis observed in PCa cells has been associated with their enhanced request for energy production, redox homeostasis, membrane formation, cell death escape, and modulation of many intracellular proliferative pathways [198,199,200,201]. Moreover, during androgen-deprivation therapy, cholesterol plays a key role in the de novo androgen synthesis, thus promoting self-sufficiency in AR signaling and hormone-refractory progression of the tumor [202,203]. Therefore, these unique metabolic features of PCa represent an optimal target for the management of this cancer. In this setting, silibinin treatment lead to the suppression of PCa aberrant lipid metabolism, both in vitro and in vivo. Mechanistically, this compound activated increased AMPK-mediated phosphorylation of sterol regulatory element-binding protein-1 (SREBP-1) and inhibited its nuclear translocation, thus reducing lipid and cholesterol accumulation and suppressing the development of androgen-independence. Moreover, the lipogenic phenotype promoted by hypoxia in PCa cells was abrogated by silibinin via inhibition of acetyl-Co A carboxylase (ACC) and fatty acid synthase (FASN) [204,205,206]. Notably, these two enzymes were also downregulated by other nutraceuticals, such as luteolin, quercetin, kaempferol, apigeninin, EGCG, and sulforaphane, in normoxic conditions [207,208,209,210].

Glutamine uptake and use is increased in various tumors, including PCa, primarily to support de novo lipogenesis. In fact, in the process of glutaminolysis, glutamine is first converted into glutamate and then into α-ketoglutarate, that can enter the Krebs cycle to drive citrate synthesis for lipogenesis [211]. Interestingly, inhibition of the glutamate-to-α-ketoglutarate conversion blocked resveratrol-related cytotoxicity in PCa cells. A similar effect was also obtained by reducing glutamine content in the culture medium, indicating that resveratrol-mediated anti-PCa effects are dependent on glutamine metabolism [212]. In addition, untargeted metabolomics and metabolic flux analysis using isotopically labeled glutamine pointed out that resveratrol in combination with ursolic acid and curcumin severely altered glutamine metabolism [213]; in particular, alanine serine cysteine transporter 2 (ASCT2) levels were found to be downregulated [213].

### 2.5. Natural Compounds Inhibiting Invasion

Metastasis, the spread of cancer cells from the primary tumor to new body tissues and organs, is a key step in PCa growth and progression [214].

During cancer development, tumor cells undergo dynamic changes leading to the acquisition of a highly invasive phenotype and to their detachment from the original tissue. Epithelial-to-mesenchymal transition (EMT) is the hallmark of this phenomenon, during which an important change in the expression of adhesion molecules regulating the interaction of tumor cells with the extracellular matrix and their microenvironment occurs. Indeed, a common characteristic of tumors of epithelial origin is an increase in the expression of N-cadherin and a parallel downregulation of E-cadherin, a major component of adherent junctions. This molecular switch is called cadherin switching, and it is generally accompanied by the upregulation of other invasion markers, such as Twist, Snail, and Slug, culminating in the enhanced metastatic potential of the tumor cell [215]. Numerous natural compounds have been shown to revert EMT in PCa cells and xenografts, particularly by modulating the PI3K/Akt and Wnt/β-catenin signaling cascades [216,217,218,219,220,221,222,223,224,225,226]. In addition, urokinase-type plasminogen activator (uPA)-, Y-box binding protein-1 (YB-1)- and SPARC/osteonectin, cwcv, and kazal-like domains proteoglycan 1 (SPOCK1)-mediated suppression of EMT contributed to the anti-invasive activity of quercetin, fisetin, and apigenin, respectively [227,228,229].

Extracellular matrix (ECM) proteolytic degradation is a key event in the metastatic process. Among more than 100 distinct proteinases, matrix metalloproteinases (MMPs) appear to be primarily responsible for most of the ECM degradation observed during metastasis [230]. In particular, MMP-2 and MMP-9 have been frequently associated with the invasiveness of tumors, including PCa. Reduction in MMP-2 and MMP-9 levels was observed after treatment with various nutraceuticals, and it generally correlates with MAPK inactivation [231,232,233,234,235,236,237,238,239,240,241,242,243,244].

One of the main events occurring during metastasis is the substitution of cell–cell interactions with integrin-based cell-matrix communication, in order to promote tumor cell invasiveness [215]. In PCa cells, silibinin treatment not only modulated the fibronectin-mediated expression of integrins (α5, αV, β1, and β3) but also induced actin remodeling and cytoskeleton disorganization via focal adhesion kinase (FAK)/Src signaling pathway inhibition [245]. Notably, disruption of microfilament-driven cell motility was also found after apigenin and curcumin treatment [246,247].

Like most tumors, PCa is characterized by CD44 dysregulation. CD44 standard (CD44s), which is present in normal epithelium, is lost in the tumor, whereas pro-invasive splice variant isoform CD44v7-10 is overexpressed [248]. CD44 inhibition is one of the mechanisms through which silibinin decreases PCa tumorigenicity. Indeed, in PC-3M cells silibinin dose-dependently reduced the mRNA and protein levels of CD44v7-10, also inhibiting early growth response protein 1 (EGR1), a regulator of CD44 promoter activity [249].

Different studies demonstrated a direct correlation between loss of metastasis suppressors/overexpression of invasion promoters and poor prognosis in human PCas. Interestingly, while genistein and EGCG induced the expression of the invasion suppressors kangai-1 (KAI1) and tissue inhibitor of matrix metalloproteinase-3 (TIMP-3) [250,251], sulforaphane-cysteine, ginsenoside Rg3, and pterostilbene inhibited the metastasis promoters galectin 1, aquaporin 1, and metastasis-associated protein 1 (MTA1) [252,253,254], respectively.

As reported above, bone metastasis commonly occurs in advanced PCas, and it responsible for considerable morbidity, such as pathologic fractures, spinal cord compression, and pain [10]. Curcumin was reported to suppress PCa bone metastasis by upregulating the invasion inhibitor bone morphogenic protein-7 (BMP-7) in vivo [255]. Bone metastasis inhibition was also observed after genistein and celastrol treatment [256,257].

### 2.6. Natural Compounds Reducing Angiogenesis

Angiogenesis, the formation of new blood vessels from preexisting capillaries, is a fundamental step in cancer development, enabling the proliferating tumor to receive oxygen and nutrients [258]. In particular, angiogenesis is characterized by the activation and migration of endothelial cells towards specific stimuli secreted by the tumor. Among several cancer-derived angiogenic factors, the most important is vascular endothelial growth factor (VEGF). The specific mitogenic effects of VEGF on the endothelial cells are mainly regulated by VEGFR-1 and VEGFR-2, two receptor tyrosine kinases. Of the two receptors, VEGFR-2 plays a fundamental role in promoting proliferation, migration, and tube formation of endothelial cells by activating multiple downstream signals, such as PI3K/Akt and MAPKs [259,260]. Interestingly, quercetin, luteolin, and celastrol at non-toxic concentrations were shown to suppress endothelial cell growth and invasion and microvessel sprouting in vitro, as well as to inhibit ex vivo angiogenesis. Mechanistically, these compounds were demonstrated to block VEGF-induced activation of VEGFR-2 and of its downstream target PI3K/Akt [261,262,263].

Hypoxia and transforming growth factor-β (TGF-β) are the two main factors implicated in the increase of VEGF secretion [264,265]. Quercetin, apigenin, and genistein were found to reduce HIF-1α expression in PCa cells, successfully preventing VEGF release [266,267,268]. Parallelly, apigenin was also shown to decrease TGF-β-induced VEGF expression by blocking the phosphorylation and nuclear translocation of Smad2 and Smad3 and by downregulating the FAK/Src/Akt pathway [269].

Thrombospondin-1 (TSP-1) is a 450 kDa extracellular calcium binding glycoprotein and a potent endogenous anti-angiogenic factor [270]. Yang et al. have recently reported that quercetin can upregulate TSP-1 mRNA and protein expression in PCa xenografts [271].

Hyaluronan is a major component of the ECM. It is a non-sulfated, linear polymer formed by repeating disaccharides of glucuronic acid (GlcUA) and N-acetyl glucosamine units (GlcNAc), and it is synthesized at the cell surface by the membrane-bound enzyme hyaluronan synthase, while being degraded by hyaluronidases [272]. In particular, the native anti-angiogenic molecule of hyaluronan can be fragmented into a smaller pro-inflammatory and pro-tumor form. Indeed, high levels of hyaluronan low-molecular-weight fragments correlate with malignant progression and poor survival in different tumor types, including PCa [273,274]. In a recent study, hyaluronan has been identified as a specific target for fisetin anti-PCa activity in tumor xenografts and TRAMP mouse models, where increased levels of anti-angiogenic high-molecular-weight hyaluronan have been found [275].

### 2.7. Natural Compounds Targeting Cancer Stem Cells

PCa stem cells (PCSCs) represent a small subpopulation of stem-like cells endowed with self-renewal and differentiation abilities, as well as with tumor-initiating and propagating functions. Expression of cell surface markers, including CD44, CD133, and α_2_β_1_ integrin, is commonly used to identify and enrich PCSCs. Owing to their resistance to standard therapies, their role in metastasis and relapse and their contribution to the progression towards castration-resistant PCa, PCSCs are currently under extensive study, especially in the field of anti-cancer drug discovery [276].

Quercetin and luteolin successfully reduced the anchorage-independent spheroid formation and the expression of CD44, ABCG2, Sox2, and Nanog in highly invasive PCa cells [277]. Moreover, while quercetin was able to block the proliferation of LNCaP- and PC3-derived CD44^+^/CD133^+^ and CD44^+^ stem cells [278], luteolin suppressed PCa stemness via upregulation of frizzled class receptor 6 (FZD6), thus inhibiting Wnt signaling [279].

Apigenin dose-dependently suppressed PCSC growth, by increasing p21 and p27 levels. In these cells, this compound also triggered extrinsic apoptosis via upregulation of TNF-α, caspase-8 and -3, and it strongly reduced invasion through downregulation of MMP-2, -9, Snail, and Slug. Furthermore, apigenin treatment induced a PI3K/Akt/NF-κB-mediated decrease in pluripotency marker Oct3/4 protein expression [280,281]. Finally, it sensitized human CD44^+^ PCSCs to cisplatin [282].

Curcumin inhibited DU145 and 22RV1-derived CD44^+^/CD133^+^ PCSC proliferation and invasion by ceRNA effect of miR-145 and lncRNA-ROR, as well as through modulation of DLK1-DIO3 imprinted gene cluster miRNAs. In fact, bioinformatic analyses and luciferase activity assays demonstrated that both the lncRNA-ROR and Oct4 mRNA contain miR-145 binding sites, and that Oct4 and lncRNA-ROR directly compete for miRNA binding. Decreasing the lncRNA-ROR endogenous levels via curcumin treatment could effectively enhance the available concentration of miR-145 in PCSCs, where miR-145 prevented cell growth by reducing Oct4 expression. Parallelly, miR-770-5p and miR-1247 expression levels were found to be significantly higher in curcumin-treated than in control PCSCs [283,284].

PCSC-like traits, including aldehyde dehydrogenase 1 (ALDH1) accelerated activity, CD49f^+^ fraction enrichment, and sphere formation capability, were abrogated by sulforaphane treatment. Notably, sulforaphane-induced suppression of PCSC-like phenotype was counteracted when c-Myc was overexpressed in PCa cells, suggesting that sulforaphane may target c-Myc-regulated PCSC-like characteristics [285].

Sphere formation was markedly suppressed after treatment of PCa cells with genistein. Moreover, treatment of PCSC-enriched spheres with genistein inhibited their growth and tumorigenicity in vivo. Additionally, this compound not only downregulated CD44 expression, but also inhibited the Hh-Gli1 pathway, which presumably contributes to the anti-CSC effect of genistein in PCa [286].

It has been reported that γ-tocotrienol could reduce CD133 and CD44 markers in castration-resistant PCa cells, also suppressing their anchorage-independent growth and spheroidogenic ability. In addition, γ-tocotrienol pretreatment of PCa cells lead tumor initiation suppression after their inoculation in nude mice. Moreover, despite being highly resistant to docetaxel, CD133^+^ cells were as responsive to γ-tocotrienol as the CD133^-^ population [287]. Similar experiments were performed by Lee et al., who confirmed the γ-tocotrienol capability to eliminate the CSC subpopulation in various PCa cell lines and mouse models, significantly inhibiting castration-resistant tumor proliferation [288]. Recent evidence indicates that also δ-tocotrienol can block PCSC growth under hypoxia via inactivation of the HIF-1α signaling [289].

## 3. Clinical Impact

To date, various clinical trials have been conducted to test the efficacy of natural compounds in PCa patients.

Two randomized, double-blind, placebo-controlled trials, aimed at evaluating the effects of curcumin on PCa patients undergoing radiotherapy, showed that this phenol could mitigate radiation-induced proctitis and oxidative stress [290,291], while six-month intake of the compound reduced the elevation of PSA in PCa men who received intermittent androgen deprivation (IAD), despite not significantly affecting the overall off-treatment duration of the therapy [292].

Accumulating epidemiological evidence has highlighted a geographical basis for PCa incidence, and isoflavone consumption may be related to this phenomenon. Indeed, PCa is more common in Western than Asian populations, and several trials have demonstrated that soy derivatives genistein and daidzein can prevent the development and progression of this tumor in Japanese and Chinese men [293,294,295,296]. On the contrary, the data collected in European patients are still contradictory. While the results obtained from two population-based case-control studies on diet, inherited susceptibility and PCa support the idea that a phytoestrogen-enriched diet may protect against the tumor in Scottish and Sicilian men [297,298], in a European Prospective Investigation into Cancer and Nutrition study genistein concentrations in the plasma samples of 1605 PCa cases and 1697 matched control participants were not correlated with cancer risk [299]. Globally, a recent meta-analysis of single patient data from seven prospective studies (two Japanese studies with 241 cases and 503 controls and five European studies with 2828 cases and 5593 controls) did not show any significant correlation between prediagnostic intake of isoflavones and PCa development, although further studies should be performed in populations where isoflavone intakes are high [300]. In this respect, it should be underlined that purified genistein have been demonstrated to be well tolerated in 20 PCa patients treated with 300 or 600 mg isoflavone/day for 84 days, showing no genotoxicity [301] and causing only minor estrogenic effects, such as hot flashes and breast changes [302].

Increased PSA serum levels are commonly observed in PCa after radical prostatectomy and are defined “biochemical recurrence” [303]. Oral administration of 60 mg/day of sulforaphane for six months, followed by two months with no treatment, led to a partial reduction of PSA levels in PCa patients who underwent prostate removal [304]. Similarly, treatment with 200 μmoles/day of sulforaphane resulted in a small (<50%) PSA decrease in patients with recurrent PCa, with a significant lengthening of the on-treatment PSA doubling time (PSADT) with respect to the pretreatment (9.6 months on-treatment vs. 6.1 months pretreatment) [305].

In a randomized placebo-controlled clinical study, PCa middle-aged men were given two doses of resveratrol (150 or 1000 mg/day) for four months: the levels of circulating androgen precursors were shown to be reduced, but no effect was observed on testosterone, DHT, and PSA levels, as well as on prostate volume [306]. In a phase I clinical trial, different doses of pulverized muscadine grape (*Vitis rotundifolia*) skin containing 4.4 μg resveratrol/500 mg extract were administered to 14 men with recurrent PCa for 2–31 months. The highest dose (4000 mg) was found to be safe and able to elongate PSADT of about 5.3 months [307]. The benefits of both the high and low (500 mg) doses were then explored in a 12-month, randomized, multicenter, placebo-controlled, phase II trial, where no changes in PSADT were evidenced in 125 patients with biochemically recurrent PCa; however, in a preplanned exploratory analysis, a significant PSADT pre-to-post increase was highlighted in patients with SOD2 Alanine/Alanine genotype (26% of total patients) treated with muscadine grape skin extract with respect to the control group, revealing the existence of a patient subpopulation which may be responsive to the treatment [308].

PCa patients scheduled for radical prostatectomy received daily doses of Polyphenon E, containing 800 mg of EGCG, until the day of surgery: serum levels of PSA, HGF, and VEGF were found to be decreased [309,310]. However, daily intake of this mixture for one year did not reduce the risk of PCa in men with high-grade prostatic intraepithelial neoplasia (HGPIN) and/or atypical small acinar proliferation (ASAP), despite being well tolerated [311]. On the contrary, positive results were obtained by treating 60 volunteers with HGPIN with 600 mg/day of EGCG: after one year, only one case of cancer was found among the 30 EGCG-treated men, while nine tumors were diagnosed among the 30 placebo-treated men. Moreover, EGCG-treated men showed lower PSA values compared to placebo-treated ones, although no significant difference was evidence between the two arms. Finally, a significant improvement of the International Prostate Symptom Score (IPSS) was observed in EGCG-treated men with benign prostatic hyperplasia [312]. In this regard, it should also be noted that PCA risk among Hong Kong and Japanese populations inversely correlates to green tea consumption and EGCG intake [313,314].

Silybin-phytosome is a commercially available formulation containing silibinin. In a phase I trial, it was orally administered to 13 patients with advanced PCa, starting from 2.5 g/day and gradually escalating to 20 g/day. No side effect was observed, except for nine cases of grade 1–2 hyperbilirubinemia. In particular, a daily dose of 13 g appeared to be well tolerated [315]. Therefore, in the subsequent study six patients with localized PCA and scheduled for prostatectomy were selected to receive three daily doses of the formulation (13 g tot), while six were chosen as controls. Silibinin blood concentrations reached a mean value of 19.7 μM after 1 h, while trough levels were 1.2 μM at the end of the 14–31 (with a mean of 20) days of treatment. On the contrary, the highest silibinin concentration observed in the prostate tissue was 496.6 pmol/g. Toxic effects were similar to those found in the previous trial. Notably, no objective PSA, IGF-I, and IGFBP-3 responses were observed in both the studies [316].

A randomized prospective double-blind study called Selenium and Vitamin E Cancer Prevention Trial (SELECT) was initiated in 2001 to determine whether vitamin E and selenium could reduce the risk of PCa in healthy men [317]. It involved more than 35,000 patients followed for up to 12 years [318]. Unfortunately, none of the tested agents, alone or in combination with each other, showed significant chemopreventive effects [318,319,320].

## 4. Conclusions

**Figure 2:**
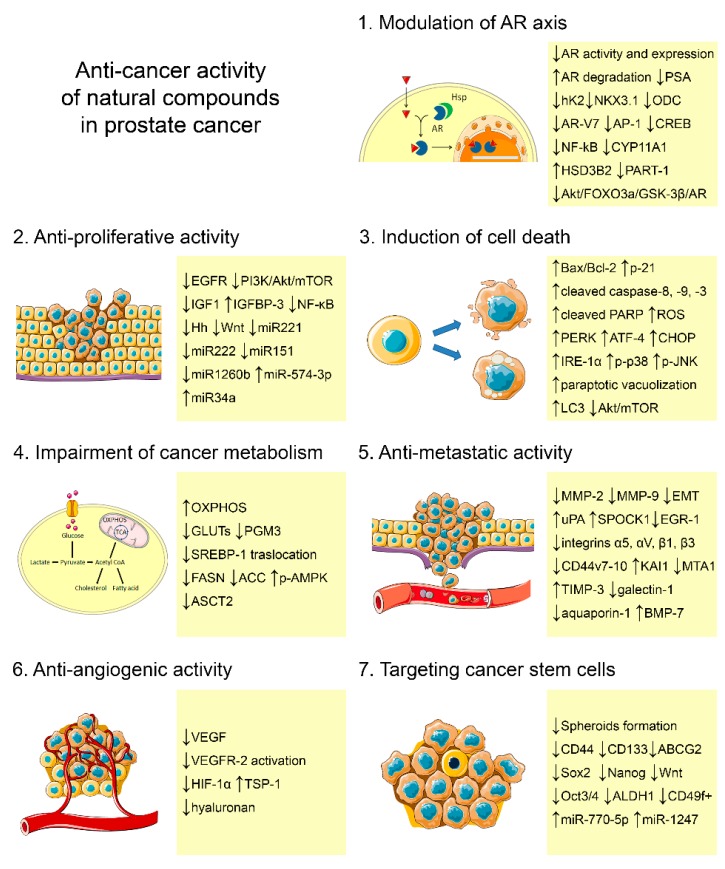
Mechanisms of action and molecular targets of phytochemicals in PCa.

This article provides an overview of recent findings about the anti-PCa activity of different natural compounds (Figure 2, Table 1).

The use of phytochemicals for PCa management offers several advantages. Firstly, natural products are safe and well tolerated, as well as usually economically affordable. Moreover, they are endowed with various in vitro and in vivo anti-tumor properties, including growth-suppressing, pro-death, anti-invasive, and anti-angiogenic activities. In particular, they appear to be able to selectively target the AR axis and the CSC subpopulation. However, these promising pleiotropic effects have been just partly confirmed in PCa patients, where nutraceutical intake has been associated with chemoprevention and PSA reduction rather than with tumor eradication. Thus, new clinical trials aimed at validating nutraceutical effectiveness in human subjects are urgently needed.

## Figures and Tables

**Table 1:** Main signaling pathways modulated by phytochemicals in PCa.

Natural Compound.	Downregulated Pathways	Upregulated Pathways	Ref.
Apigenin	PI3K/Akt, Hh axis Proteosomal activity Glucose uptake Invasion/Motility Angiogenesis PCa cell stemness	Extrinsic apoptotic cell death in PCa stem cells	[91,92,112,193,217,229,246,267,268,269,280,281,282]
Berberine	AR axis EGFR levels and activity Invasion/Motility	Apoptotic cell death Programmed-necrotic cell death	[73,148,149,168,169,225]
Celastrol	AR axis Proteosomal activity Oncogenic miRNAs Invasion/Motility Bone metastasis Angiogenesis	ER stress Apoptotic cell death Paraptotic cell death Autophagic cell death	[75,126,156,161,176,188,226,257,263]
Curcumin	AR signaling Testosterone levels PI3K/Akt, Hh, Wnt axis Oncogenic miRNAs Glutaminolysis Invasion/Motility Bone metastasis PCa cell stemness	ER stress Apoptotic cell death ROS production Programmed-necrotic cell death Oncosuppressive miRNAs	[42,43,44,45,93,94,107,111,122,132,133,134,158,161,162,170,213,218,233,234,235,247,255,283,284]
EGCG	AR signaling PI3K/Akt, Hh axis Lipogenesis Invasion/Motility	Apoptotic cell death	[66,67,98,107,145,209,241,242,243,251]
Fisetin	AR stability and function Invasion/Motility Angiogenesis	Autophagic cell death	[35,185,228,232,275]
Genistein	AR signaling EGFR levels and activity PI3K/Akt, NFκB, Hh, Wnt axis Oncogenic miRNAs Proteosomal activity Lipogenesis Invasion/Motility Bone metastasis PCa cell stemness	Apoptotic cell death Oncosuppressive miRNAs	[56,57,58,59,60,61,81,95,96,101,102,107,112,115,116,117,118,119,120,140,154,155,208,219,220,236,237,238,239,240,250,256,286]
Ginsenosides	AR axis NFκB signaling Oncogenic miRNAs Metastasis promoters	Oncosuppressive miRNAs	[68,69,70,71,125,253]
Honokiol	AR axis	Apoptotic cell death	[74,153]
Kaempferol		Apoptotic cell death	[131]
Luteolin	AR signaling EGFR levels and activity IGFR signaling Oncogenic miRNAs Proteosomal activity Lipogenesis Endothelial cell growth Microvessel sprouting PCa cell stemness	Apoptotic cell death Oncosuppressive miRNAs	[38,79,88,121,130,207,262,277,279]
Quercetin	AR signaling AR-V7 activity EGFR levels and activity Pi3K/Akt, Hh, Wnt axis Lipogenesis Invasion/Motility Endothelial cell growth Microvessel sprouting PCa cell stemness	ER stress Apoptotic cell death	[28,29,30,31,32,77,78,90,110,127,128,129,157,161,216,227,231,261,266,271,277,278]
Resveratrol	AR signaling EGFR levels and activity NFκB, Hh signaling Oncogenic miRNAs Glucose fermentation Glutaminolysis	ER stress Apoptotic cell death ROS production Autophagic cell death Mitochondrial oxidation Oncosuppressive miRNAs	[47,48,49,50,51,52,53,54,80,100,107,123,124,135,136,137,138,139,161,163,164,186,190,191,212]
Sibilinin	IGF1 expression Wnt cascade Lipogenesis and lipid-dependent metabolism Invasion/Motility	ER stress Apoptotic cell death ROS production	[72,85,86,87,113,147,159,161,204,205,206,222,223,224,245,249]
Sulforaphane	AR function PI3K/Akt, NFκB axis Glycolysis Penthose Phosphate shunt Lipogenesis and lipid-dependent metabolism Invasion/Motility Metastasis promoters PCa cell stemness	Apoptotic cell death ROS production	[62,63,64,65,97,103,141,142,143,144,165,166,195,196,197,210,221,252,285]
Tannic acid		ER stress Apoptotic cell death	[160,161]
Tocotrienols	NFκB signaling PCa cell stemness	ER stress Apoptotic cell death Paraptotic cell death Autophagic cell death	[105,161,175,287,289]
Ursolic acid	NFκB signaling Glutaminolysis	Apoptotic cell death	[104,150,152,213]
